# N6-Methyladenosine-Regulated mRNAs: Potential Prognostic Biomarkers for Patients With Lung Adenocarcinoma

**DOI:** 10.3389/fcell.2021.705962

**Published:** 2021-08-06

**Authors:** Junjun Sun, Yili Ping, Jingjuan Huang, Bingjie Zeng, Ping Ji, Dong Li

**Affiliations:** Department of Laboratory Medicine, Shanghai Tongji Hospital, School of Medicine, Tongji University, Shanghai, China

**Keywords:** lung adenocarcinoma, N6-methyladenosine, prognostic signature, multi-omics, biomarkers

## Abstract

Aberrant regulation of m6A mRNA modification can lead to changes in gene expression, thus contributing to tumorigenesis in several types of solid tumors. In this study, by integrating analyses of m6A methylation and mRNA expression, we identified 84 m6A-regulated mRNAs in lung adenocarcinoma (LUAD). Although the m6A methylation levels of total RNA in LUAD patient tumor tissue were reduced, the majority (75.2%) of m6A-regulated mRNAs were hypermethylated. The m6A-hypermethylated mRNAs were mainly enriched in terms related to transcription factor activity. We established a 10-m6A-regulated-mRNA signature score system through least absolute shrinkage and selection operator Cox regression analysis, with its predictive value validated by Kaplan–Meier curve and time-dependent receiver operating characteristic curves. RFXAP and KHDRBS2 from the signature also exhibited an independent prognostic value. The co-expression and interaction network analyses demonstrated the strong correlation between m6A regulators and the genes in the signature, further supporting the results of the m6A methylation modification patterns. These findings highlight the potential utility of integrating multi-omics data (m6A methylation level and mRNA expression) to accurately obtain potential prognostic biomarkers, which may provide important insights into developing novel and effective therapies for LUAD.

## Introduction

Lung adenocarcinoma (LUAD) is the most common histological subtype of lung cancer and shows high morbidity and mortality rates as well as poor prognosis (Ma et al., 2020). In the past two decades, the understanding of the molecular pathogenesis of LUAD has greatly expanded, and research has identified gene expression patterns, diagnostic markers and therapeutic targets of LUAD (Boneva et al., 2020). However, there is still an urgent need to provide more effective biomarkers for the early diagnosis, prognosis and monitoring of LUAD.

N6-methyladenosine (m6A) is the most abundant post-transcriptional mRNA modification and regulates the alternative splicing, localization, and stability of mRNAs, therefore influencing the level of subsequent protein expression (Huang et al., 2020). m6A modification of mRNAs is dynamically regulated by m6A RNA methylation regulators, including methyltransferases (METTL3, METTL14, METTL16, and WTAP), demethylases (FTO and ALKBH5), and binding proteins (YTH-domain containing proteins, HNRNP, and IGF2BP1-3) (Bauer et al., 2020). Studies have shown that aberrant regulation of m6A modification of mRNAs contributes to the development of lung cancer (Zhu et al., 2020b). Expression of m6A RNA methylation regulators is closely associated with lung cancer growth (Guo et al., 2018; Bai et al., 2019; Wanna-Udom et al., 2020), and recent reports have indicated that some m6A regulators may serve as prognostic markers in LUAD (Sletvold et al., 1988; Zhang et al., 2020; Zhu et al., 2020c). Morever, m6A modification on some key genes mRNA could influence the key genes expression that are involved in cancer cell progression, including carcinogenesis, proliferation, and metastasis (Gao et al., 2020). Thus, abnormalities in mRNA methylation are promising diagnostic markers for the early detection and prognosis of cancer. However, most studies have focused on exploring the prognostic significance of m6A methylation regulators (Zhao et al., 2020), and the transcriptome-wide m6A modification profile of mRNAs in LUAD have not been well explored.

Recent advances in methylated immunoprecipitation sequencing (MeRIP-Seq, or m6A-seq) have made it possible to efficiently profile the transcriptome-wide distribution of m6A in diverse human tissues and cell types (Wang et al., 2017). Using integrated analyses of m6A methylation and gene expression, studies have reported highly diverse m6A modification patterns in tumor cells compared with non-tumor adjacent tissues in gastric cancer (Sang et al., 2020) and colorectal cancer (Zhang et al., 2021). Although several studies have examined the expressions, potential functions, and prognostic values of m6A methylation regulators, fewer reports have investigated the prognostic value of genes regulated by m6A methylation. Therefore, a comprehensive analysis of m6A methylation and gene expression may offer a new avenue of targets and strategies for LUAD diagnosis and treatment.

Here, we characterized the transcriptome-wide m6A modification profile in LUAD tissues and analyzed the correlation between m6A modification and gene expression. We selected m6A-regulated mRNAs and performed univariate Cox regression analyses of m6A-regulated gene expression in LUAD. We developed a potential risk signature to determine whether m6A-regulated genes could serve as potential prognostic biomarkers of LUAD.

## Materials and Methods

### Tissue Samples

Six pairs of LUAD tissues (three stage I LUAD tissues and three stage III LUAD tissues) and paired adjacent non-tumor tissues for microarray study were collected from the Department of Otorhinolaryngology at the Shanghai Tongji Hospital. The clinicopathological characteristics of the study subjects are summarized in Supplementary Table 1. All patients have not undergone any treatment before surgery. Written informed consent was obtained from all patients prior to enrollment in the present study. The study was approved by the Medical Ethics Committee of Shanghai Tongji Hospital.

### m6A RNA Methylation Quantification

Total RNA was extracted from tumor tissues with Trizol reagent (Life Technologies, United States). m6A methylation was detected and quantified using the Abcam m6A RNA Methylation Quantification Kit (Abcam, United Kingdom), following the manufacturer’s protocol. The m6A content was quantified by reading the absorbance of samples at 450 nm according to the standard curve.

### Real-Time PCR Analysis and MeRIP qPCR

Total RNA was isolated using TRIzol reagent (Invitrogen, United States) following the manufacturer’s protocol. cDNA was synthesized by reverse transcription using PrimeScript II 1st strand cDNA Synthesis Kit (Takara, Japan). Total RNA (3 μg) was added to 300 μL 5 × IP buffer (50 mM Tris HCl pH 7.4, 750 mM NaCl, 0.5% IGEPAL CA-630) containing 2 μg affinity purified anti-m6A rabbit polyclonal antibody (Synaptic Systems, Germany), and the samples were incubated in a rotary shaker for 2 h at 4°C. Rabbit anti-human IgG antibody (Abcam, United Kingdom) was used as negative control. The samples were then mixed with 20 μL sheep anti-rabbit IgG magnetic beads (Invitrogen, United States) blocked in advance with 0.5% BSA for 2 h. The mixture was incubated at 4°C for 12 h. The microtubes were placed on a magnetic separator for 2 min. The supernatant containing RNA without m6A modification was transferred to another tube as the “supernatant” sample. The beads were washed three times with 300 μL 1× IP buffer and twice with 300 μL 1× wash buffer (100 mM Tris HCl pH 7.4, 50 mM NaCl, 0.1% IGEPAL CA-630). The m6A-modified RNA was eluted from the magnetic beads by 300 μL elution buffer (100 mM Tris HCl pH 7.4, 1 mM EDTA, 0.05% SDS, 4 μL proteinase K, 2 μL RNase inhibitor) in the rotary shaker for 2 h at 50°C and then transferred to a tube as the “IP” sample. The RNA was extracted by phenol:chloroform:isoamylol (25:24:1) reagent. Each sample was analyzed using RT-qPCR. Real-time PCR was performed using SYBR^®^ Premix Ex Taq^TM^ II (Takara, Japan) on the AB 7500 Real time PCR system machine (AB Applied Biosystems, United States). PCR primer sequences used for qPCR are listed in Supplementary Table 2. The relative expression levels of mRNA were quantified using the ΔΔCt method. The m6A modification level of RNA was calculated using the following formula.

$$\%\mathrm{Input}=\frac{2^{-\mathrm{CtIP}}}{2^{-\mathrm{CtIP}}+2^{-\mathrm{CtSupernatant}}}\times 1\times 100\%$$

### Western Blot Analysis

Cells were lysed in cell lysis buffer (Cell Signaling Technology, United States) supplemented with protease and phosphatase inhibitors (Roche, Germany). Protein samples (30 μg) were separated by 10% SDS polyacrylamide gels and blotted onto Hybond-C Extra membranes (Amersham Bioscience, United Kingdom). Primary antibodies for western blot analysis included the following: rabbit anti-METTL3, rabbit anti-ALKBH5, rabbit anti-RFXAP (Proreintech, China), rabbit anti-KHDRBS2 (Absin,China) and rabbit anti-β-actin (Cell Signaling Technology, United States). HRP-conjugated goat anti-rabbit (Merck Millipore, United States) was used as secondary antibody.

### Arraystar Human m6A Epitranscriptomic Microarray Analysis

Human m6A epitranscriptomic microarray and mRNA microarray were obtained from Arraystar Company (Rockville, MD, United States). Briefly, the total RNAs were immunoprecipitated with anti-m6A antibody. The elution from the immunoprecipitation magnetic beads was used as the “IP” sample. The recovered supernatant was used as the “Sup” samples, and Labels “IP” and “Sup” RNA were used for Cy5 and Cy3, respectively. After merging, it was hybridized to the Arraystar Human m6A Epitranscriptomic Microarray (8 × 60 K). An Agilent scanner G2505C was used to scan the array. The “m6A methylation level” was calculated was the percentage of modification based on the IP (Cy5-labeled) and Sup (Cy3-labeled) normalized intensities. The raw data of microarray have been uploaded to GEO database (GSE176348).

### Overview of Differentially m6A-Methylated mRNAs and Gene Ontology Analysis

The differentially m6A-methylated mRNAs between LUAD and paired adjacent non-tumor tissues were calculated for fold change (| log2(FC)| > 0.5) and statistical significance by unpaired *t*-test (*p* ≤ 0.05) and graphically represented on volcano plots and heatmap. Gene ontology (GO) analysis associates the differentially m6A-methylated mRNAs enriched in certain gene ontological functions and GO terms.^1^ Pathway analysis associates the differentially m6A-methylated mRNAs enriched in certain biological pathways. The statistical significance of the enrichment was calculated by the Fisher Exact test *p*-value and also -log10(*p*) transformed as the enrichment score.

### Integration of m6A Methylation and mRNAs Expression Data

The “mRNA expression level” was calculated based on the total of IP (Cy5-labeled) and Sup (Cy3-labeled) normalized intensities of RNA. The differential expression of each mRNA was calculated for fold change (| log2(FC)| > 2) and statistical significance by unpaired *t*-test (*p* ≤ 0.05). Differentially m6A-methylated RNAs or differentially expressed RNAs between two comparison groups were identified by filtering with the fold change and statistical significance (*p*-value) thresholds.

### Public Databases and Analysis

For establishing the risk model, the entire cohort of patients with LUAD (*N* = 468) from TCGA was randomly divided into a training set (*N* = 236) and a validation set (*N* = 232) to construct and assess the prognostic model. The clinicopathological characteristics of the training set and validation set are summarized in Supplementary Tables 3, 4. The R package “glmnet” (Friedman et al., 2010) was used to perform a least absolute shrinkage and selection operator (LASSO) Cox regression (iteration = 1000) to penalize a m6A-regulated mRNA prognostic signature for LUAD patients that can stratify the patients into high-risk and low-risk groups. The risk score calculation formula is:

$$Riskscore=\sum_{i=1}^{n}Coefi*x_{i}$$

where Coef_*i*_ means the coefficients, and x_*i*_ is the expression value of each m6A-regulated mRNAs. The Kaplan–Meier log-rank test (R package “tsurvival”), time-dependent ROC analysis (R package “timeROC”), univariate and multivariate analyses were performed to evaluate the predictive ability of the prognostic m6A-RPS. Spearman’s correlation analysis was used to evaluate the association between m6A methylation regulators and the 10 prognostic m6A-regulated mRNAs and plots visualized with R package “corrplot.” Gene co-expression networks were constructed with R package “reshape2” and visualized with R package “igraph.” The related R codes were uploaded to the git-hub repository. All statistical analyses were analyzed using the R Programming Language version 3.6.1 and SPSS statistics software version 24.0.

## Results

### Reduced m6A Level in LUAD

**FIGURE 1:**
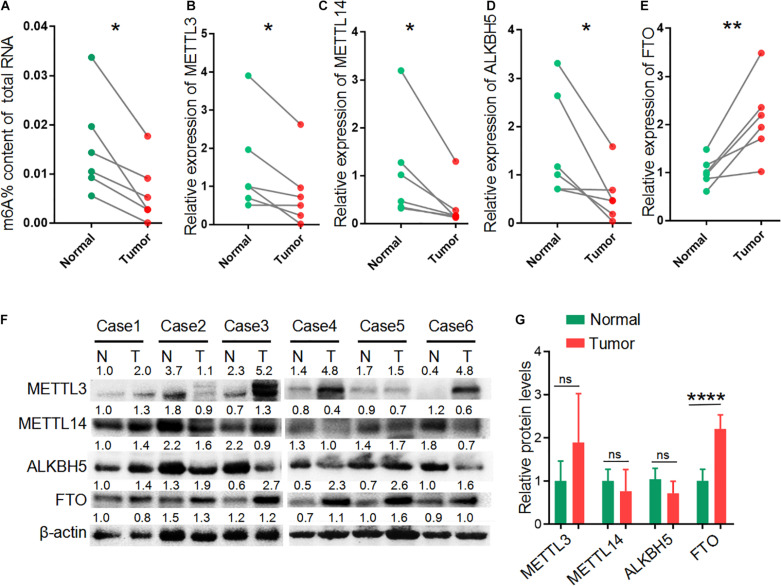
Reduced m6A level in LUAD. **(A)** The global m6A level of total RNAs in six paired LUAD patient tumor tissues (Tumor) and matched adjacent non-tumor tissues (Normal). **(B–E)** The relative expression of METTL3, METTL14, ALKBH5 and FTO mRNA in six paired LUAD tissues compared with those in adjacent non-tumor tissues. **(F)** Western blot showing the protein expression of METTL3, METTL14, ALKBH5 and FTO in LUAD tissues (T) and adjacent non-tumor tissues (N). **(G)** Relative protein levels calculated based on the band density in western blot results. *P < 0.05, **P < 0.01, and ****P < 0.0001.

We performed transcriptome-wide analyses of m6A-methylated mRNAs and overall mRNA levels in six pairs of LUAD tissues and adjacent non-tumor tissues by microarray analyses. We also quantified the global m6A level in total RNAs in LUAD tissues relative to control samples. All six tumor samples showed a decrease in global m6A levels compared with the non-tumor samples (*p* = 0.0101) (Figure 1A). Because the dynamic equilibrium of m6A level is regulated by methyltransferases and m6A demethylases, we next examined the RNA and protein levels of methyltransferases (MELLT3 and METTL14) and demethylases (ALKBH5 and FTO). The expression of MELLT3, METTL14 and ALKBH5 mRNA were decreased in LUAD tissues compared with matched normal adjacent lung tissues (Figures 1B–D), while differences were not observed at the protein level (Figures 1F,G). Demethylase FTO was increased significantly in LUAD tissues compared normal tissues both in mRNA and protein levels (Figures 1E–G). These results suggest that the decreased m6A levels in the LUAD tissues may be from increased levels of the FTO demethylase.

### Overview of Differentially m6A-Methylated mRNAs in LUAD

**FIGURE 2:**
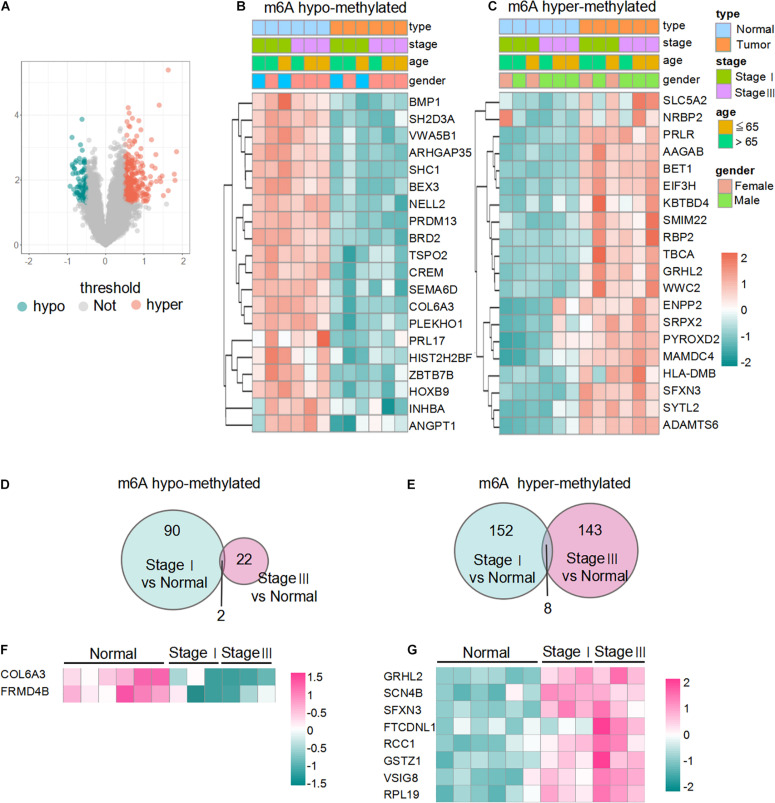
Overview of differentially m6A-methylated mRNAs in LUAD. **(A)** Volcano plot of differentially m6A-modified mRNAs based on log2FC > 0.5 and *p*-value < 0.05. **(B,C)** Heatmaps of the top 20 m6A hypo- and hypermethylated mRNAs in LUAD. Data are shown in green (m6A hypomethylation) to red (m6A hypermethylation). **(D,E)** Venn diagrams showing the overlapping hypo- and hypermethylated mRNAs between stage I samples and stage III samples. **(F,G)** Heat-maps of the two hypomethylated mRNAs and eight hypermethylated mRNAs between stage I samples and stage III samples.

A total of 255 mRNAs, including 194 hypermethylated and 61 hypomethylated mRNAs, showed significant differences in m6A methylation levels in LUAD (*p* < 0.05, | log2(FC)| > 0.5) (Figure 2A). To validate the findings of microarray analysis, MeRIP-qPCR and qPCR were performed on three randomly selected hypermethylated mRNAs and three hypomethylated mRNAs on the same RNA samples used in microarray analysis. The m6A methylation and mRNA expression of the selected genes were consistent with the data from the microarray results (Supplementary Figure 1). The top 20 m6A hypermethylated and hypomethylated mRNAs are shown in Figures 2B,C. The results revealed 90 hypomethylated mRNAs and 152 hypermethylated mRNAs in stage I LUADs, 22 hypomethylated mRNAs and 143 hypermethylated mRNAs in stage III LUADs (Figures 2D,E). There were only two hypomethylated mRNAs and eight hypermethylated mRNAs were common to both stage I LUAD and stage III LUAD tissues (Figures 2F,G). This may be that the methylation of m6A is dynamically regulated and affected by depend on diverse conditions and this may also be caused by random fluctuations in samples included into the study.

### Functional Enrichment of Differentially m6A Methylated mRNAs

**FIGURE 3:**
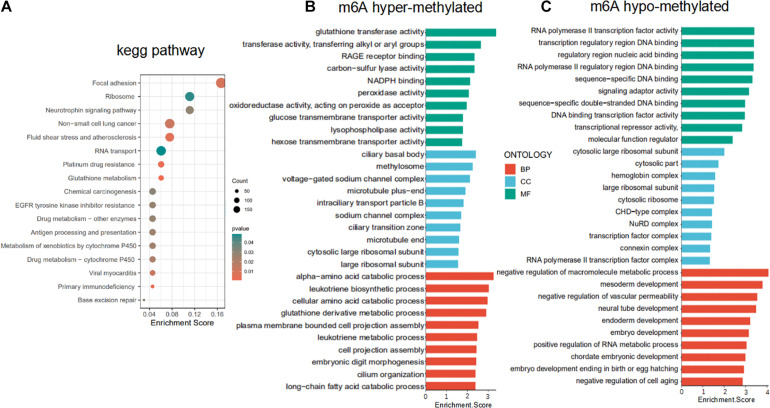
Functional enrichment of differentially m6A methylated mRNAs. **(A)** KEGG pathway enrichment analysis of hypo- and hypermethylated mRNAs. **(B,C)** Gene ontology (GO) enrichment analysis of the m6A hypermethylated and hypomethylated mRNAs.

To uncover the biological pathways potentially affected by differential m6A methylation of mRNAs, all m6A hypo- and hypermethylated mRNAs were uploaded to DAVID database to identify overrepresented KEGG pathways. The results of KEGG pathways analysis suggested that abnormal m6A methylation plays an important role in focal adhesion, RNA transport, non-small cell lung cancer, and EGFR tyrosine kinase inhibitor resistance (Figure 3A). GO analysis revealed that m6A hypermethylated mRNAs were enriched in oxidoreductase activity (glutathione derivative metabolic process, NADPH binding, peroxidase activity, and oxidoreductase activity) and tnutrient metabolism process (cellular amino acid catabolic process, glucose and hexose transmembrane transporter activity). Whereas the m6A hypomethylated mRNAs were enriched in biological processes GO terms were related to RNA biosynthetic processes (RNA polymerase II transcription factor activity and RNA metabolic process) and developmental process (embryo development, mesoderm development, and neural tube development) (Figures 3B,C). Our data again validate that the broad role of m6A-motif mRNAs in regulating important cellular pathways and processes in LUAD.

### Integration of m6A Methylation and mRNA Expression Data to Obtain m6A-Regulated mRNAs

**FIGURE 4:**
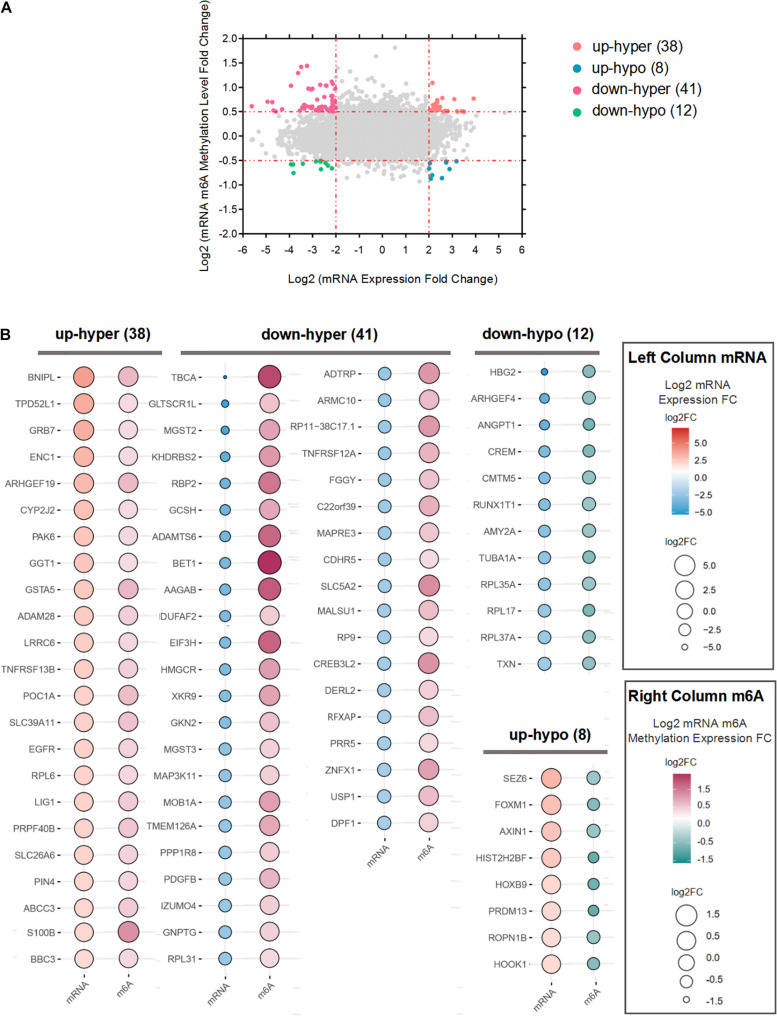
Integration of m6A methylation and mRNA expression data to obtain m6A-regulated mRNAs. **(A)** Four-quadrant plots to show the mRNAs with a significant change both in m6A and mRNA levels. **(B)** Bubble plot showing the mRNA expression levels and m6A modification levels of m6A-regulated mRNAs.

We performed a combination analysis of the 255 differentially m6A methylated mRNAs with their expression level (*p*-value < 0.05, | log2(FC)| > 2). A total of 84 mRNAs that were both differentially m6A methylated and expressed in LUAD tissues were classified into four categories based on expression (upregulated/downregulated) and methylation (hypo/hypermethylation) (Figure 4A). Among the 84 mRNAs, the majority (75.2%) mRNAs with up (23; up-hyper) or down (41; down-hyper) regulated were related to hyper m6A methylation. While 20 m6A hypomethylated mRNAs were significantly upregulated (8; up-hypo) or downregulated (12; down-hypo) (Figure 4B). We defined the genes whose expression level is significantly correlated with m6A methylation level as the m6A-regulated genes. Based on these data, we ultimately obtained 84 candidate m6A-regulated mRNAs.

### Evaluation of the Prognostic Performance of the m6A-Regulated mRNAs

**FIGURE 5:**
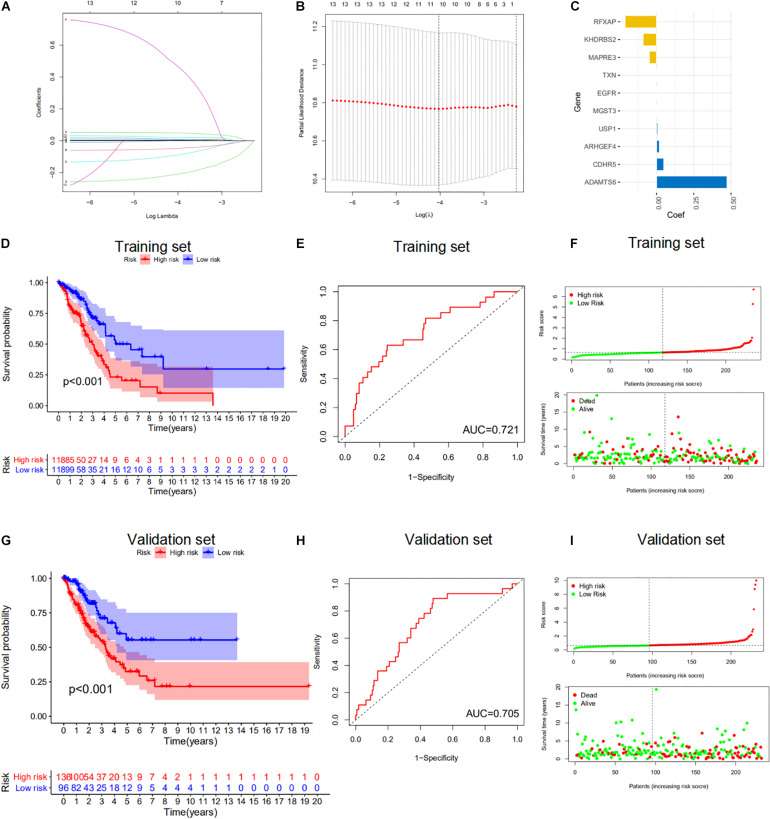
Evaluation of the prognostic performance of the m6A-regulated mRNAs. **(A–C)** Least absolute shrinkage and selection operator (LASSO) regression was performed to calculate the minimum criteria **(A,B)** and coefficients **(C)**. **(D,G)** Kaplan–Meier survival analysis of high-risk (red) and low-risk (blue) LUAD patients in the training set and validation set. **(E,H)** The receiver operating characteristic curve (ROC) analysis for the prognosis prediction of the signature of overall survival (OS) in the training set and validation set and the area under curve (AUC) was calculated. **(F,I)** Distributions of risk scores and survival status of LUAD patients in the training set and validation set.

We next explored the clinical significance of the 84 m6A-regulated mRNAs. We performed univariate Cox proportional-hazards regression analyses to identify m6A-regulated mRNAs that correlated with the overall survival (OS) of LUAD patients (Figures 5A,B). We then used LASSO Cox regression analysis to select 10 genes with the best prognostic value to build a m6A-regulated-mRNA signature (m6A-RPS) in the TCGA cohort. The risk score for each patient was calculated based on the expression level of the 10 genes according to the model coefficients (Figure 5C). Using the median risk scores, patients with LUAD from the TCGA cohort were divided into high- and low-risk subgroups. Kaplan–Meier survival curves showed that patients with high-risk score showed worse survival outcomes in the training set and validation set (Figures 5D,G). ROC curve analyses demonstrated that the m6A-RPS could predict OS in patients with LUAD (training set, AUC = 0.721; validation set, AUC = 0.705) (Figures 5E,H). The distributions of the risk score were plotted along with the corresponding survival outcome (Figures 5F,I).

### Prognostic Analysis of the m6A-RPS

**FIGURE 6:**
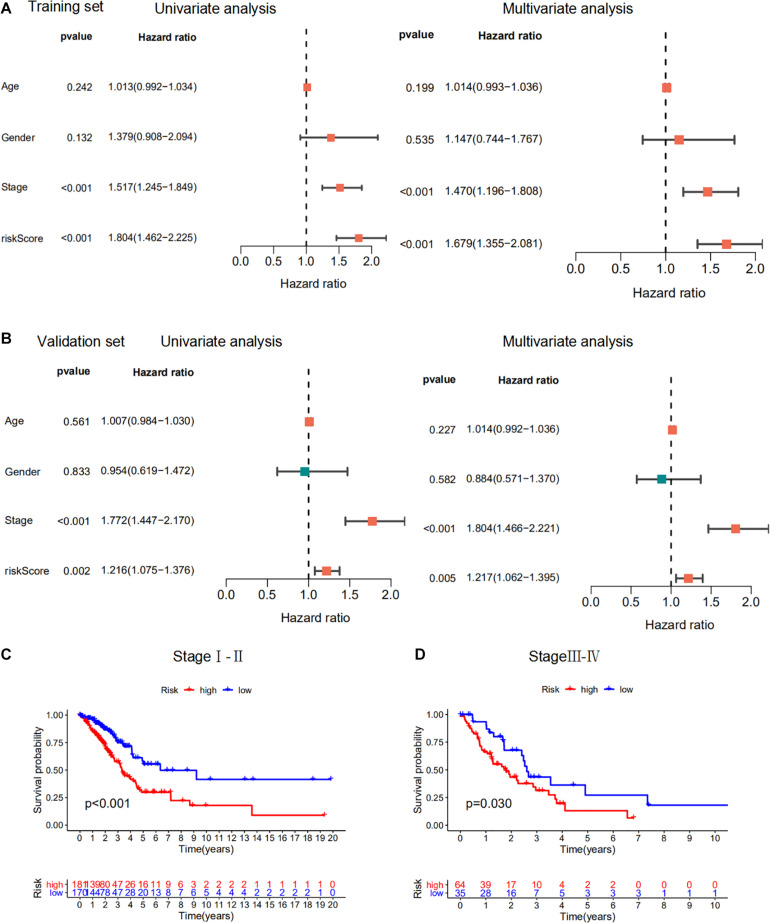
Prognostic analysis of the m6A-RPS. **(A,B)** Univariate and multivariate Cox regression analysis of correlations between risk score for m6A-RPS and clinical parameters in the training set and validation set. **(C,D)** Kaplan–Meier survival analysis of m6A-RPS prognostic value in LUAD stage I–II and stage III–IV cases in the TCGA cohort.

We performed univariate and multivariate Cox regression analysis to evaluate the prognostic value of the m6A-RPS and other clinicopathological features in the training dataset (Figure 6A). The results showed that m6A-RPS was remarkably associated with tumor stage (HR = 1.517, 95% CI: 1.245–1.849; *p* < 0.001). The performance of m6A-RPS was also tested in the validation set (Figure 6B). Similar results were observed in early stage (stage I–II) and advanced stage (stage III–IV) patients. High-risk patients tended to have a worse prognosis than low-risk patients (Figures 6C,D). Together, these data indicate that m6A-RPS was an independent prognostic factor for LUAD patients and might be useful for clinical prognosis evaluation.

### Principal Component Analysis

**FIGURE 7:**
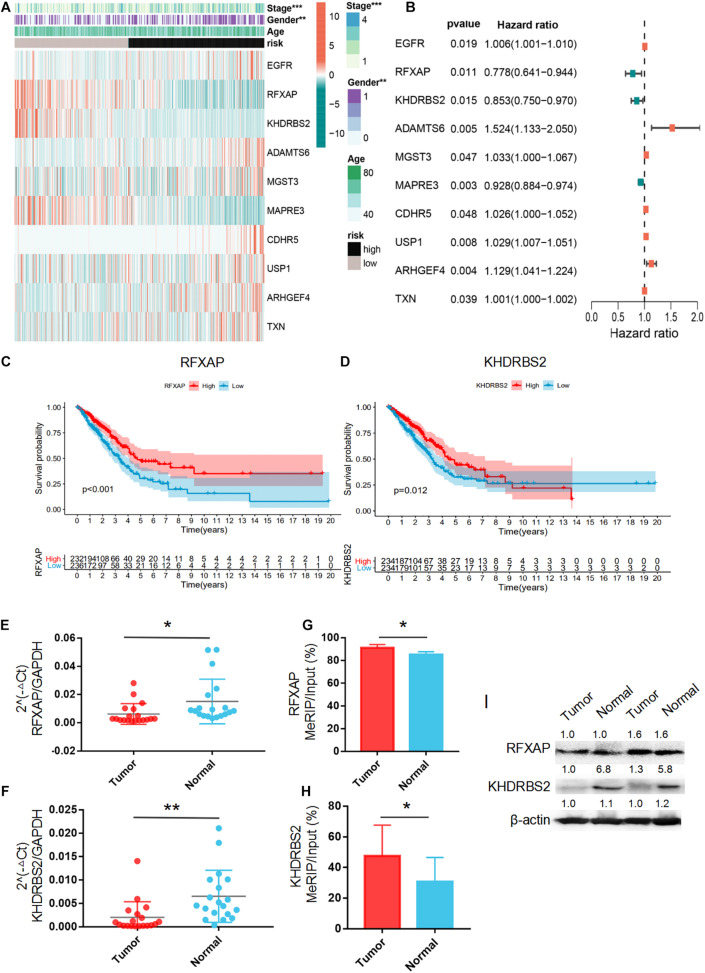
Principal Component Analysis. **(A)** Forest plot of the prognostic ability of the 10 m6A-regulated mRNAs included in the prognostic signature. **(B)** Heatmap of the 10 m6A-regulated mRNA expression levels with different clinicopathological features. **(C,D)** Overall survival curves according to RFXAP and KHDRBS2 mRNA expression in TCGA LUAD patient cohorts. The mRNA expression level **(E,F),** m6A methylation level **(G,H)** and protein expression level **(I)** of RFXAP and KHDRBS2 in LUAD in LUAD tissues (Tumor) and adjacent non-tumor tissues (Normal). *P < 0.05, **P < 0.01, and ***P < 0.001.

The heatmap of the expression levels of the 10 prognostic m6A-regulated mRNAs in high- and low-risk LUAD patients in the TCGA dataset revealed that the expression levels of RFXAP, KHDRBS2, and MAPRE3 mRNAs decreased with increasing risk score, while the expression levels of ADAMTS6, MGST3, ARHGEF4, and TXN mRNAs were associated with increased risk scores (Figure 7A), which was consistent with the results of LASSO Cox regression analysis (Figure 7B). Kaplan–Meier survival curves confirmed that higher expression of RFXAP and KHDRBS2 mRNAs were associated with better OS in the TCGA dataset (Figures 7C,D). We further verified the expressions of RFXAP and KHDRBS2 mRNAs in 19 LUAD patients, the results showed that RFXAP and KHDRBS2 expressions in tumor tissues were lower than that in matched normal adjacent lung tissues (Figures 7E,F), which were consistent with that of GEPIA database (Supplementary Figure 2). Since m6A modifications play a key role in the modulation of translation, we associatively analyzed the m6A methylation levels and protein expression of RFXAP and KHDRBS2. The MeRIP-qPCR and WB results showed that although the m6A methylation levels of KHDRBS2 and RFXAP were both increased in tumor tissues (Figures 7G,H), the protein expression levels of KHDRBS2 was decreased, while RFXAP protein expression was unchanged (Figure 7I).

### Co-expression and Interaction Network Analyses Between m6A Regulators and m6A-RPS

**FIGURE 8:**
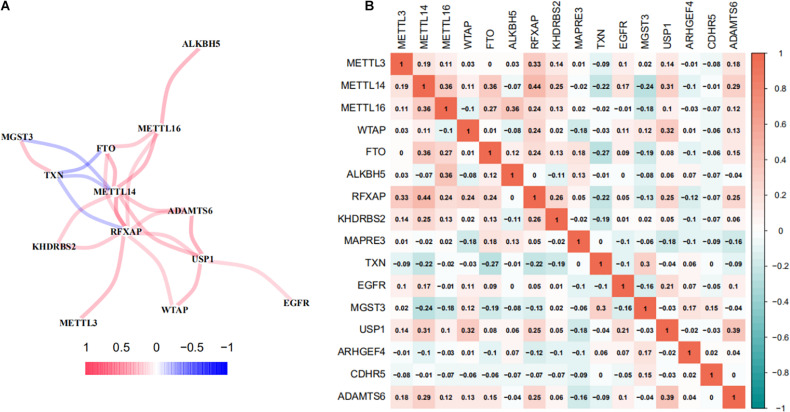
Co-expression and interaction network analyses between m6A regulators and m6A-RPS. **(A)** Spearman correlation analysis and **(B)** co-expression network of m6A RNA methylation regulators, methyltransferases (METTL3, METTL14, METTL16, and WTAP), demethylases (FTO and ALKBH5) and 10 m6A-regulated mRNAs in the prognostic signature in TCGA LUAD patient cohorts.

A co-expression network analyses was performed to investigate the interactions between m6A-RPS and m6A methylation regulators. As shown in Figure 8A, m6A methylation regulators METTL14, METTL16, and FTO were shared as hub genes in more than one module. There was a highly significant positive correlation between m6A-RPS (RFXAP, ADAMTS6, KHDRBS2, USP1) and m6A methylation regulators METTL14, METTL16, and FTO. MGST3 and TXN were negatively related to m6A methylation regulators METTL14 and FTO. We also performed a Spearman correlation analysis to investigate the relationship between the m6A methylation regulators and the 10 prognostic m6A-regulated mRNAs. Consistent with the results of co-expression network analyses, the expression levels of RFXAP, KHDRBS2, USP1, and ADAMTS6 mRNAs were significantly correlated with the levels of methyltransferase (METTL3, METTL14, METTL16) and FTO demethylase mRNA expression in TCGA database (Figure 8B).

## Discussion

Lung cancer remains the leading causes of cancer-related death worldwide, and LUAD is the most common subtype of lung cancer. m6A. The most frequent modification of eukaryotic mRNA, has recently been reported to correlate with the progression of various cancers (Cheng et al., 2021), but its role in LUAD is not yet clear. In this study, we characterized the transcriptome-wide m6A modification profile in LUAD tissues and analyzed the correlation between m6A modification and gene expression. We selected m6A-regulated mRNAs and performed univariate Cox regression analyses of m6A-regulated gene expression in LUAD. Ten m6A-regulated genes were used to establish an m6A-RPS for predicting the OS of LUAD patients. Patients with LUAD from the TCGA cohort were divided into high- and low-risk subgroups using median risk scores, and we found that patients with high-risk score showed worse survival outcomes. Univariate and multivariate Cox regression analyses confirmed that m6A-RPS was an independent risk factor for OS. Finally, the co-expression and interaction network analyses demonstrated the strong correlation between m6A regulators and m6A-RPS expression, further supporting the results of the m6A methylation modification patterns.

The m6A modifications on mRNA affects almost every stage of mRNA metabolism, from mRNA stability, translation efficiency, splicing and localization, all of these are closely connected to protein expression. We identified 194 hypermethylated and 61 hypomethylated protein coding genes in LUAD. The majority mRNAs with increased or decreased expression were related to hyper m6A methylation. Prior research reports that transcripts with more m6A modifications tend to be less stable and influencing the splicing kinetics of nascent pre-mRNAs (Wang et al., 2014; Louloupi et al., 2018; Liang et al., 2020). However, follow-up studys found that the m6A modificatied mRNA can either promote or inhibit translation, depending on the m6A reading protein “reader” (Leonetti et al., 2020; Xu et al., 2020). Notably, we observed that most genes with m6A methylation level changes were not accompanied by significant gene expression level changes. This may be because m6A modification not only regulates mRNA expression but also regulates mRNA structure and interactions between mRNA and specific binding proteins (Zhu et al., 2020a). Furthermore, m6A methylation is dynamic, which m6A motifs are methylated depending on diverse conditions (Wu et al., 2016; Yan et al., 2018). So experimental validations are nevertheless needed to further verify our findings.

Emerging evidence suggests that m6A RNA modifications are related to tumorigenesis, invasion, and metastasis. However, the diversity of m6A regulators and m6A modified genes was strikingly different. Whether the m6A modified gene can be used as a prognostic marker is unknown. In the present study, a 10-m6A-regulated-gene signature (EGFR, RFXAP, KHDRBS2, ADAMTS6, MGST3, MAPRE3, CDHR5, USP1, ARHGEF4, and TXN mRNA) was identified as an independent prognostic factor for OS in LUAD patients. RFXAP and KHDRBS2 from our signature exhibited an independent prognostic value in LUAD. RFXAP (regulatory factor X-associated protein) is a critical transcription factor for MHC II molecules and may also bind other tumor suppressor proteins (Ding et al., 2015). KHDRBS2 has been reported to link tyrosine kinase signaling cascades with aspects of RNA metabolism (Wang et al., 2002) and overexpression of KHDRBS2 was related to better overall survival in LUAD (Li et al., 2020). We obvious that although the KHDRBS2 and RFXAP mRNAs were both decreased with the increased m6A methylation levels, the protein expression levels of KHDRBS2 was decreased, while RFXAP protein expression was unchanged. These results suggested that m6A methylation is not the only determinant for gene expression, the regulatory function of m6A in mRNA remains to be fully explored.

Our study revealed the features of transcriptome-wide distributions of m6A modifications of mRNAs in LUAD and presented the relationship between m6A methylation extent and the transcript level. And a 10-m6A-regulated-gene signature was identified as an independent prognostic factor for OS in LUAD patients. There are several limitations to our research, in particular the small number of specimens used, which may have affected the values of the data presented. And our 10-m6A-regulated-gene prognostic model should be validated using more independent cohorts and biological experiments. We hope that our results may help to identify the prognostic m6A-regulated mRNAs, thereby providing insights into their potential roles in LUAD tumorigenesis and progression.

## Data Availability Statement

The datasets presented in this study can be found in online repositories. The names of the repository/repositories and accession number(s) can be found below: GEO, GSE176348.

## Ethics Statement

The studies involving human participants were reviewed and approved by Medical Ethics Committee of Shanghai Tongji Hospital. The patients/participants provided their written informed consent to participate in this study.

## Author Contributions

DL and PJ constructed the study and were responsible for drafting the manuscript. PJ, JS, and YP performed the data analysis and plotted the figures. BZ and JH did the polymerase chain reaction experiments. All authors read and approved the final manuscript.

## Conflict of Interest

The authors declare that the research was conducted in the absence of any commercial or financial relationships that could be construed as a potential conflict of interest.

## Publisher’s Note

All claims expressed in this article are solely those of the authors and do not necessarily represent those of their affiliated organizations, or those of the publisher, the editors and the reviewers. Any product that may be evaluated in this article, or claim that may be made by its manufacturer, is not guaranteed or endorsed by the publisher.

## Footnotes

1
http://www.geneontology.org

## Supplementary Material

Supplementary Figure 1 **(A)** The expression levels of six selected mRNAs in six paired LUAD patient tumor tissues (Tumor) and matched adjacent non-tumor tissues (Normal). **(B)** The m6A methylation level of six selected mRNAs in six paired LUAD patient tumor tissues (Tumor) and matched adjacent normal tissues (Normal).

Supplementary Figure 2 The mRNA expression level of RFXAP **(A)** and KHDRBS2 **(B)** in GEPIA datebase.
