# Supplementary figures and images for: N6-Methyladenosine-Regulated mRNAs: Potential Prognostic Biomarkers for Patients With Lung Adenocarcinoma
Source: Front Cell Dev Biol. 2021 Aug 6;9:705962. doi: 10.3389/fcell.2021.705962 (PMC8377381; doi:10.3389/fcell.2021.705962)

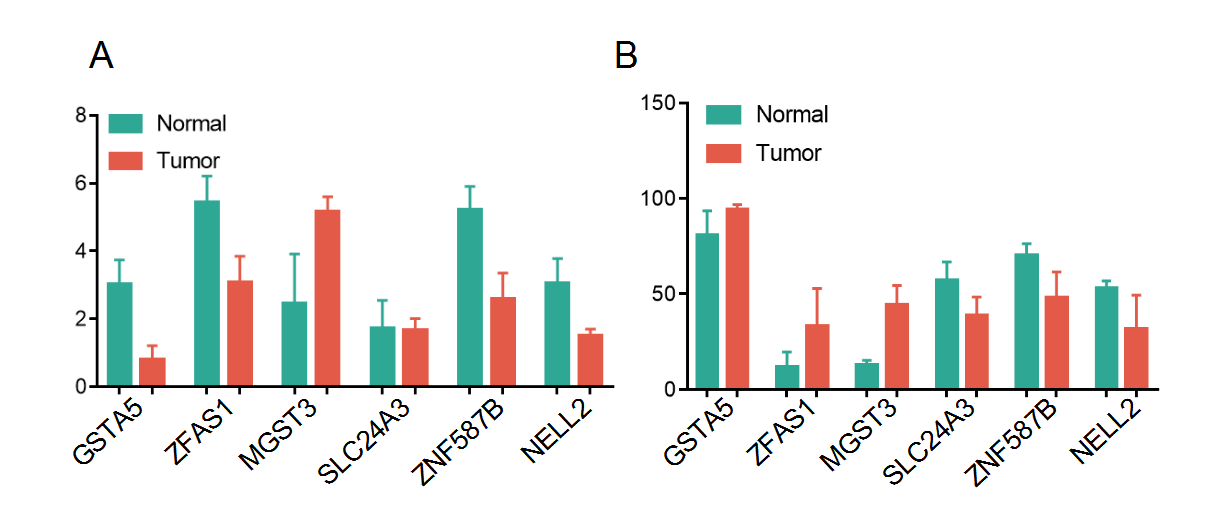

Supplement: Supplementary Figure 1 — (A) The expression levels of six selected mRNAs in six paired LUAD patient tumor tissues (Tumor) and matched adjacent non-tumor tissues (Normal). (B) The m6A methylation level of six selected mRNAs in six paired LUAD patient tumor tissues (Tumor) and matched adjacent normal tissues (Normal). [file Image_1.TIF]

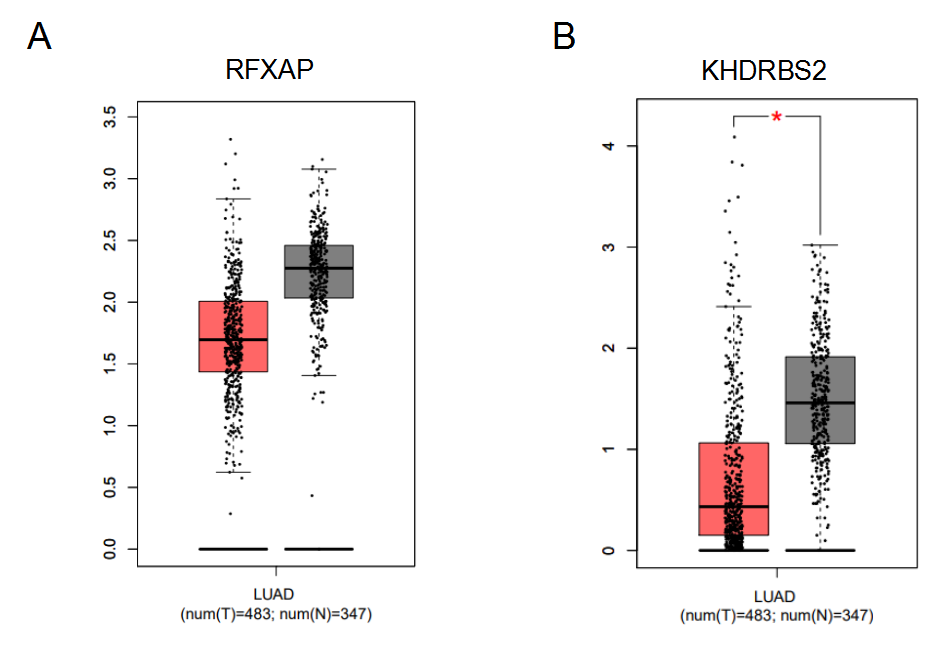

Supplement: Supplementary Figure 2 — The mRNA expression level of RFXAP (A) and KHDRBS2 (B) in GEPIA datebase. [file Image_2.TIF]
